# Examination of risk exposure models during COVID-19 in relation to youth life satisfaction and internalizing symptoms

**DOI:** 10.1038/s41598-022-20661-2

**Published:** 2022-09-28

**Authors:** Romy S. Snetselaar, Juliëtte M. Liber, Suzanne M. Geurts, Ina M. Koning

**Affiliations:** 1 Department of Developmental Psychology, Utrecht University, Utrecht, The Netherlands; 2 Department of Interdisciplinary Social Science, Utrecht University, Utrecht, The Netherlands

**Keywords:** Human behaviour, Environmental impact

## Abstract

This study examined mediation of a negative COVID-impact on the relationship between risk exposure, and life satisfaction and internalizing symptoms in youth (aged 9–18). Four operationalizations of risk exposure were applied; an Additive versus a Cumulative Risk Model (ARM and CRM), risk clusters and the most salient risk factors. Results showed that a stronger negative COVID-impact is related to lower life satisfaction, more internalizing symptoms and higher additive and cumulative risk. ARM and CRM’s effect on lower life satisfaction is mediated through negative COVID-impact, though not for internalizing symptoms. Clusters of risk factors and risk factors within clusters significantly related to a stronger negative COVID-impact are the clusters ‘Individual factors’ (low self-control), ‘Parenting’ (negative mother–child interaction and low parental responsiveness), ‘Maternal mental health’ and ‘Demographic factors’ (low SES and high paternal education). From all significant risk factors, low self-control, low parental responsiveness, negative mother–child interaction and low SES were most salient.

## Introduction

Late 2019 COVID-19 started to spread throughout the world arriving in the Netherlands in early 2020. Most countries took measures to slow the exponential spread of COVID. The Dutch government implemented a lockdown, as of March 15th, 2020 which entailed the closure of all childcare institutions and schools (unless parents held ‘crucial’ professions), as well as establishments in the leisure and food service industry^1^. Additionally, social distancing and staying home were promoted.

Preliminary research showed more loneliness and depressive symptoms among adolescents during the COVID-19 pandemic and other negative effects on youth’s mental and social health^2,3^. The negative effects of the pandemic on psychological wellbeing appeared not only related to the virus itself, but also to the necessary restrictions and safety measures^4^. For some, restrictions resulted in an accumulation of losses, e.g. school closures entailed the loss of access to a variety of resources such as school mental health care and peer groups, all increasingly important during adolescence for psychological well-being^5,6^. Subsequentially, the lockdown and COVID-19 restrictions may have increased the number and intensity of risk factors to which youth and families are exposed. Risk factors, or stressors, refer to individual or environmental factors that are associated with an increased risk of negative (developmental) outcomes^7^. For some youth, risk factors tend to cluster together and the likelihood to be exposed to a broad assortment of physical and psychological stressors is higher, f.e. those in poverty or with ethnic minority/migration backgrounds^8,9^. The lockdown-related restrictions may have caused an increase in risk factors and a decrease in (access to) protective factors against risk exposure. For example, with the loss of (physical) education, a large group of youth who spent most of their time at home could have become more dependent on their already burdened family^10^. With the accumulation of stressors, physiological response systems are overwhelmed, self‐regulatory coping processes are disrupted, resilience decreases and the likelihood of serious (developmental/psychological) problems in youth is greater than expected based on the sum or additive effect of the individual risk factors^8,11,12^. The effect of the (non-exponential) sum of risk factors is called Additive Risk Model (ARM). The effect of the exponential accumulation of risk is referred to as the Cumulative Risk Model (CRM)^9,13^. Both risk models were expected to negatively affect youth well-being in the COVID-19 situation.

In the current study, we investigated the impact of additive and cumulative risk exposure on the perceived negative impact of COVID-19 (negative COVID-impact) and its subsequent relation with youth well-being, operationalized as life satisfaction and internalizing symptoms. Additive and cumulative risk exposure were expected to be related to a stronger negative COVID-impact, and a stronger negative COVID-impact was, in turn, expected to be related to lower psychological well-being for youth. It was hypothesized that both additive as well as cumulative risk were related to a stronger negative COVID-impact and level of youth well-being. However, since CRM implies an exponential growth model, rather than simply a sum or linear growth model, cumulative risk was expected to better account for the negative associations than additive risk.

In addition, to identify the most salient risk factors, the relationships between individual risk factors and clusters of risk factors with negative COVID-impact were mapped as well. Included were 5 categories containing interrelated risks (risk clusters): **Individual factors** (*educational level, self-control, social competence*) are important predictors of psychological wellbeing^14–16^. **Parenting factors** (*quality of the parent–child interaction, parental responsiveness and frequency and duration of parent–child activities*) are also important predictors of (un)favorable youth development and wellbeing^17–19^. **Maternal mental health** & **paternal mental health** (*stress, depression* and *anxiety*) are consistently related to youth wellbeing, as meta-analytic findings show^20^. **Family constellation** (*household size, parent–child ratio, family composition*), relates to youth wellbeing as well^3,21,22^. Lastly, **Demographic family factors** (*family SES, migration background, parental educational level*)*,* are consistently important for youth developmental outcomes^22–24^.

## Method

### Procedure

This cross-sectional study used data from the first measurement of the Digital Family project: a Dutch, ongoing longitudinal research project on digital media use in the family context. Families with at least one child aged 9–18 years were recruited through different channels; social media, Utrecht University media, school newsletters, personal communications, etc. Interested parents completed an application form including contact information. Then, parents received login information and informed consent for themselves and their children < 16 years. At the start of the online questionnaire, participants were informed again about the study and asked to sign active consent. Data were collected in April-July 2020, coinciding with lockdown restrictions. The ethics committee of Utrecht University granted ethical approvement (FETC-20-192). All methods were performed in accordance with the relevant guidelines and regulations.

### Participants

The research sample consisted of 395 parents (with *M*_age_ = 46.65, *SD* = 5.27) of which 58.2% were mothers. In the data file parents within the same family were labeled as Parent 1 (P1, mothers; N = 229, 100% female) and Parent 2 (P2, fathers; N = 166, 99.4% male, since one family consisted of 2 mothers). Four hundred youths (M_age_ = 13.49, *SD* = 2.14, range 9–18 years) participated, of which 53.3% were girls. In total, the data represented 487 unique parent–child combinations. Most youth (69.3%) attended secondary school at the time of the data collection. Nearly all parents and youth (95%) had a Dutch ethnic background. Of all participating youth, 67.9% attend Senior general secondary, or pre-university education and 69.9% of parents attended Higher professional education or University. See Table 1 for all descriptives.

**Table 1:** Descriptives (sample size, mean and standard deviation) for all variables.

Category	Variables	N	M	SD	Min	Max
Main/outcome variables	Negative COVID-impact	400	21.01	4.40	11	33
	Life Satisfaction	392	7.65	1.24	2	10
	Internalizing symptoms^1^	400	1.15	0.85	0	3
	Cumulative risk	260	9.98	15.41	0	100
	Additive risk	260	2.42	2.03	0	10
Individual factors	Educational level	277	4.66	1.60	1	6
	Self-control	392	28.72	5.12	13	43
	Social competence	395	20.46	3.83	5	25
Parenting	Negative interaction with P1	397	3.42	1.47	2	12
	Negative interaction with P2	397	3.36	1.82	2	12
	Parental responsiveness	395	11.94	1.82	7	15
	Frequency of joint activity	397	31.79	5.56	14	46
	Duration of joint activity	397	4.43	1.43	1	7
Maternal mental health	Stress P1	374	6.09	1.99	4	16
	Depression P1	371	2.56	0.93	2	7
	Anxiety P1	374	3.08	1.26	2	8
Paternal mental health	Stress P2	287	5.73	1.72	4	12
	Depression P2	287	2.67	0.96	2	7
	Anxiety P2	287	2.85	1.24	2	8
Family constellation	Household size	389	4.38	0.96	2	7
	Parent–child ratio	389	1.41	0.61	0.50	5
Demographic factors	SES	393	12.38	1.74	6	15
	Educational level P1	375	4.65	0.69	1	5
	Educational level P2	289	4.46	0.98	1	6

P1 = mothers & P2 = fathers.

For family composition and migration background, see the “Measures” section.

### Measures

In the selection of measures in the original study, the original aim of that study (digital media use) was taken into account, as well as the composition of a questionnaire with a total number of items that parents and youth could fill out within a limited time frame. Therefore, sometimes subscales of lengthier validated measures were included.

#### Youth reports

*Life satisfaction* was measured using the Cantril Ladder of Life Satisfaction^25^, that allowed the participants to rate their current life between 0 and 10 with ‘0’ being the worst possible life and ‘10’ being the best possible life. This measure is a single-item Likert-scale previously used in the Health Behavior and School Children study (HBSC.org) and validated in several countries^26^.

*Internalizing symptoms* were measured with the Patient Health Questionnaire (PHQ-4^27^, Dutch version), a 4-item commonly used and validated^28^ screening instrument for anxiety and depressive symptoms on a 4-point Likert scale (1 = not at all to 4 = (nearly) every day), asking how often feelings occur in the past two weeks (f.e. “I felt nervous, anxious or restless”, “I felt down, depressed or hopeless”). A sum score (range = 4–16) was recoded into: 0 = ‘no symptoms’ (= 4), 1 = ‘mild symptoms’ (5 or 6), 2 = ‘moderate symptoms’ (7–10), 3 = ‘severe symptoms’ (11–16) (Cronbach’s α = 0.717 in the current study).

*Negative impact of the COVID-19 pandemic and its restrictions* was measured by nine items formulated by researchers at Utrecht University (see Appendix A), that addressed fear, home atmosphere, activity and sleep. Items like “Because of the COVID-crisis there are more conflicts within the family” were rated on a 5-point Likert scale (‘completely disagree’ = 1 to ‘completely agree’ = 5). The original included 11 items, two items were excluded since they did not address a potential negative COVID-impact (e.g. I adhere as closely as possible to the COVID regulations.) If applicable items were recoded to ensure that a higher sum score indicated a more negative COVID-impact (Cronbach’s α = 0.544 in the current study).

*Self-control* was assessed using the 5-item self-control measure^29,30^ (Dutch version) rated on a 5-point Likert scale (‘not true at all’ = 1 to ‘completely true’ = 5). Example items are ‘I have trouble saying no’ and ‘I do certain things that are bad for me, if they are fun’. A higher sum score reflected higher self-control (Cronbach’s α = 0.617 in the current study).

*Social competence* was measured using the 5-item Dutch version of the Harters’ Self Perception Profile of Adolescents^31^. We used the subscale “Close Friendships” for assessing the ability to establish and retain close friendships (range ‘not true at all’ = 1, to ‘completely true’ = 5). An example item is “I find it hard to get friends on whom I can count.” Items were recoded so that a higher sum score indicated a higher level of perceived social competence (Cronbach’s α = 0.666).

*Negative parent–child interaction* was measured with two items derived from the ‘conflict’ and ‘antagonism’ subscales each from the ‘Negative interaction’ scale from the ‘Network of Relationship Inventory’ (NRI^32^). This total measure and its subscales were included and validated in previous studies^33^. An example item is ‘How often do you and your mother disagree or are arguing?’ Items were rated along a 6-point Likert scale (‘little or none’ = 1 to ‘the most’ = 5 and 6 = parent is deceased). A sum score was calculated for the perceived interactions with fathers (Cronbach’s α = 0.885 in current study) and mothers (Cronbach’s α = 0.761 in current study) separately.

*Parental responsiveness* was measured with a Dutch translation form a commonly used scale from the ‘Parenting Style Inventory’ (PSI-II;^34^) including three items rated on a 5-point Likert scale (‘completely disagree’ = 1 to ‘completely agree’ = 5). An example item is ‘I know my parents are there for me when I’m in trouble’. A higher sum score reflected more parental responsiveness (Cronbach’s α = 0.627 in the current study).

*Frequency of parent–child joint activity* was measured using a ten-item measure about the frequency of different parent–child joint activities in the past two weeks, on a 6-point Likert scale ('not at all' = 1 to ‘(nearly) every day’ = 6)^35^. Example activities are ‘having dinner’, ‘going out for a walk’, ‘talk about things’. A higher sum score indicated more parent–child joint activities (Cronbach’s α = 0.622 in the current study).

*Duration of parent–child joint activity* was measured with a single item about how much time parents and children spent together during the past two weeks doing different parent–child joint activities, with response options ranging from less than 5 min (= 1) to more than 4 h (= 7). This measure was newly developed in a previous study (LEF;^36^), not yet validated.

#### Parent reports

*Depression* (two items) *and anxiety* (two items)*,* were measured among mothers and fathers separately using the ‘Patient Health Questionnaire’ (PHQ-4^27^, Dutch version). An example item for anxiety is ‘I felt nervous, anxious or restless’. In addition, for parental stress a similar brief and commonly used measure was included consisting of four items of the stress-subscale of the Depression Anxiety Stress Scale 21 (DASS-21^37^). All items were rated on a 4-point Likert scale (‘not at all’ = 1 to ‘(nearly) everyday’ = 4). The three subscales were computed by calculating the sum score of the items, where a higher score indicated higher levels of depression, anxiety and parental stress (Cronbach’s α’s ranged from 0.702 to 0.828 in the current study).

#### Demographics

Demographic characteristics previously identified as developmental risk factors were coded for the current study as 0 (no risk) or 1 (risk). Variables included the following:

For *migration/ethnic background*, being of Dutch background was coded as ‘0’ no risk and common minority nationalities were ‘1’ risk. *Educational level of youth* was coded as ‘0’ no risk (Senior general secondary education, Pre-university education, Secondary vocational education, Higher professional education) and ‘1’ risk (primary school or multiple levels of Pre-vocational secondary education). *Parental highest achieved educational level* was codes as ‘0’ no risk (Secondary vocational education, Higher professional education and University education) and ‘1’ risk (Primary school, Pre-vocational secondary education, Senior general secondary education, Pre-university education-no secondary education). *Socioeconomic status* was measured among parents with four items from the ‘Family Affluence Scale’^38^ and was calculated by the sum score of the items, with a higher score indicating a higher socio-economic status. *Household size* reflected the number of adults and youths in the household. *Parent–child ratio* was computed by dividing the number of children in the home by the number of parents in the home. A higher score thus indicated a more unfavorable parent–child ratio. This factor was separated from household size, because the household size might appear typical, while the ratio can be unfavorable. For *family composition* parents were assigned no-risk (0 = 2-parent families) and risk (1 = single parent families due to divorce, death or not in the picture).

#### Additive and cumulative risk exposure

A cumulative risk index has typically been operationalized as the additive summation of dichotomized risk factors^9^. However, the term “Cumulative” implies an exponential growth model, rather than simply a sum or linear growth model which is used under the same name in many studies. For that reason, the current study challenged the status quo by introducing a distinction between ‘additive’ and ‘cumulative’ risk. Additive risk being a sum score of dichotomized risk factors (previously/typically referred to as cumulative risk) and cumulative risk being the squared score of that sum to account for an exponential growth effect, which has previously been found in studies differentiating between ‘cumulative risk’ and ‘squared cumulative risk’^39^. How these scores were obtained is discussed below.

To obtain a score for Additive risk, a sum of all abovementioned risk factors was computed. To do this, T-scores were used based on composite scores of the scales/risk factors. Generally, ratings of T < 40 and T > 60 are considered a deviation from the typical population (corresponding with deviating from the norm by at least 1 standard deviation). Thus, scores surpassing T60 or below T40 were considered risk (0 = no risk & 1 = risk). For example, for responsiveness T < 40 was considered a risk and for parental depression T > 60 was a risk. Then, all risk scores were added up to create an Additive risk score. This additive score was squared to obtain a Cumulative risk score. Cases where no parent-data are available were excluded for the ARM & CRM scores, due to then missing parental and environmental risk factors.

### Data-analysis

Data were analyzed in IBM's SPSS Statistics Version 25^40^. First, descriptive statistics and intercorrelations were reported. Spearman correlations were obtained as amongst other variables, CRM and ARM were non-normally distributed. Then, the relationship of negative COVID- impact with life satisfaction and internalizing symptoms was tested with linear regression analyses.

Mediation effects of ARM and CRM on life satisfaction and internalizing symptoms through negative COVID-impact were examined by consecutive multiple regression analyses: First, the effect of ARM on negative COVID impact, internalizing symptoms and life satisfaction were mapped in separate regression analyses, then the mediator (negative COVID-impact) was added to the regression model to see how the effect of ARM on life satisfaction and internalizing symptoms changed. If the effect of COVID-impact on life satisfaction or internalizing symptoms was significant and the effect of ARM significantly attenuated in this hierarchical model, this suggested that the effect of ARM on life satisfaction /internalizing symptoms was mediated through negative COVID-impact on youth^41^. In sum, three models were used to test mediation effects, and repeated for either ARM or CRM as independent variable and internalizing symptoms or life satisfaction as dependent variable.

Next, a stepwise approach was applied to further investigate the risk clusters in relation to negative COVID-impact. The five clusters were regressed separately on negative COVID-impact. Lastly, the significant individual risk factors emerging from each cluster were included in another linear regression model with negative COVID-impact to identify the most salient risk factors that remain.

## Results

### Correlations

Table 2 shows an overview of correlations among variables of interest and the correlations of the risk factors with these variables of interest.

**Table 2:** Spearman correlations for youth reported (outcome) variables (1–5) and correlations for outcome variables with risk factors.

Variables		1	2	3	4	5
1. Negative COVID-impact		–				
2. Life Satisfaction		− 0.406**	–			
3. Internalizing symptoms^a^		0.444**	0.456**	–		
4. Cumulative risk		0.230*	− 0.187*	0.066	–	
5. Additive risk		0.230**	− 0.187*	0.066	1.000**	–
Individual factors	Educational level	0.007	− 0.039	0.134*	− 0.272***	− 0.272***
	Self-control	− 0.387***	0.406***	− 0.402***	− 0.261***	− 0.261***
	Social competence	− 0.193***	0.151**	− 0.049	− 0.273***	− 0.273***
Parenting	Negative interaction with P1	0.255***	− 0.224***	0.194***	0.325***	0.325***
	Negative interaction with P2	0.121*	− 0.087	0.139**	0.177***	0.177***
	Parental responsiveness	− 0.160***	0.111*	− 0.124*	− 0.229***	− 0.229***
	Frequency of joint activity	− 0.104*	0.123*	− 0.032	− 0.307***	− 0.307***
	Duration of joint activity	− 0.158**	0.135**	− 0.092	− 0.294***	− 0.294***
Maternal mental health	Stress P1	0.114*	0.013	0.105*	0.094	0.094
	Depression P1	0.055	− 0.077	0.039	0.194***	0.194***
	Anxiety P1	0.155**	− 0.087	0.083	0.163***	0.163***
Paternal mental health	Stress P2	0.066	− 0.012	0.072	0.372***	0.372***
	Depression P2	0.087	− 0.105	0.006	0.291***	0.291***
	Anxiety P2	0.062	0.042	0.081	0.286***	0.286***
Family constellation	Household size	0.005	0.102*	− 0.011	0.147*	0.156*
	Parent–child ratio	0.081	0.002	0.037	0.247**	0.247**
	Family composition	0.047	− 0.067	0.015	0.187**	0.187**
Demographic factors	SES	− 0.144**	0.094	0.008	− 0.301***	− 0.301***
	Migration background	0.045	0.142*	− 0.030	0.143*	0.143*
	Educational level P1	− 0.002	0.094	0.063	− 0.322***	− 0.322***
	Educational level P2	0.052	0.026	0.212***	− 0.159**	− 0.159**

Asterisks signify significant effects, *p < 0.05, **p < 0.01 & ***p < 0.001.

^a^Recoded (1 = no symptoms, 2 = mild symptoms, 3 = moderate symptoms, 4 = severe symptoms).

### COVID-19 impact and youth wellbeing

Negative COVID-impact predicted a significant 17.2% of the variability in life satisfaction (*B* = − 0.117, 95% CI [− 0.142, − 0.091], β = − 0.414, *R*^2^ = 0.172, adjusted *R*^2^ = 0.170, F (1,390) = 80.832, *p* = 0.000). It also predicted a significant 19.8% of variability in youths internalizing symptoms (*B* = 0.086, 95% CI [0.069, 0.103], β = 0.444, *R*^2^ = 0.198, adjusted *R*^2^ = 0.195, F (1,398) = 97.959, *p* = 0.000).

Additionally, an independent samples *t* test revealed that negative COVID-impact was larger for girls (*N* = 215, *M* = 21.74, *SD* = 4.52) than for boys (*N* = 185, *M* = 20.15, *SD* = 4.103), with a mean difference of 1.598, 95% CI [− 2.452, − 0.744], *t*(398) = − 3.679, *p* = 0.000, two-tailed (small-medium effect: Cohen’s *d* = 0.368). Regression analysis revealed a significant positive relationship between age and negative COVID-impact (β = 0.112, *p* = 0.025).

### Mediation of ARM & CRM on youth wellbeing through negative COVID-19 impact

**Figure 1:**
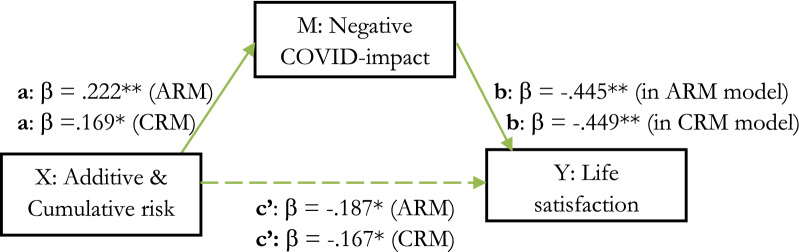
Mediation effects. ARM/CRM → Negative COVID-impact → Life satisfaction. *Note.* Green arrow = significant, dashed = significant in separate model, but non-significant when negative COVID-impact is added to the model; *p < 0.01 & **p < 0.001.

First, additive risk significantly predicted a portion (3.5%) of the variability in life satisfaction (c’: *R*^2^ = 0.035, adjusted *R*^2^ = 0.031, *F* (1,258) = 9.362, *p* = 0.002, small effect: *f*^2^ = 0.036). Then, additive risk significantly related to negative COVID-impact (a: *R*^2^ = 0.049, adjusted *R*^2^ = 0.046, *F* (1,258) = 13.406, *p* = 0.000, small effect: *f*^2^ = 0.05). But, when negative COVID-impact was added to the same regression model, additive risk was no longer a significant predictor for variability in life satisfaction (*p* = 0.109), whereas negative COVID-impact was (b: *R*^2^ = 0.223, adjusted *R*^2^ = 0.217, *F* (2,257) = 36.850, *p* = 0.000), with a medium to large effect for the full (combined) hierarchical model (*f*^2^ = 0.287). Thus, the effect of additive risk on life satisfaction seems to be (fully) mediated through negative COVID-impact. The model accounted for a significant 22% of the variability in life satisfaction. With regard to the CRM, abovementioned analyses yielded similar results (c’: *R*^2^ = 0.029, adjusted *R*^2^ = 0.025, *F* (1,258) = 7.616, *p* = 0.006, *f*^2^ = 0.030, small effect, a: *R*^2^ = 0.028, adjusted *R*^2^ = 0.024, *F* (1,258) = 7.411, *p* = 0.007, *f*^2^ = 0.029, small effect & b: *R*^2^ = 0.224, adjusted *R*^2^ = 0.217, *F* (2,257) = 36.987, *p* = 0.000, *f*^2^ = 0.289, medium-large effect). Results are presented in Fig. 1.

**Figure 2:**
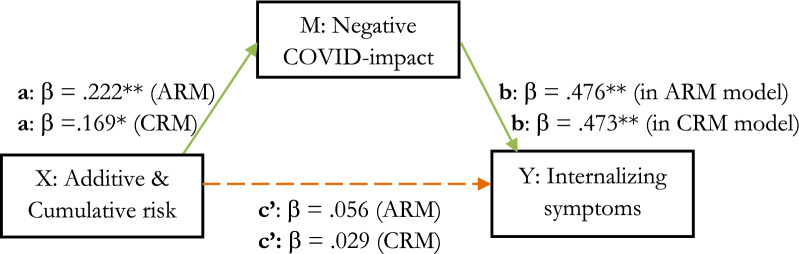
Mediation effects. ARM/CRM → Negative COVID-impact → Internalizing symptoms . *Note.* Green arrow = significant, orange dashed arrow = non-significant in separate model and still non-significant when negative COVID-impact is added to the model; *p < 0.01 & **p < 0.001.

The analyses were repeated with internalizing symptoms as outcome variable. Additive risk was not significantly related to internalizing symptoms (c’: *R*^2^ = 0.003, adjusted *R*^2^ = 0.001, *F* (1,258) = 0.799, *p* = 0.372), neither after adding negative COVID-impact to the model (*p* = 0.376). However, additive risk related significantly to negative COVID-impact (a: *R*^2^ = 0.049, adjusted *R*^2^ = 0.046, *F* (1,258) = 13.406, *p* = 0.000, small effect: *f*^2^ = 0.05, predicting 4.9% variability) and negative COVID-impact accounted for a significant 21.8% of variability in internalizing symptoms (b: *R*^2^ = 0.218, adjusted *R*^2^ = 0.212, *F* (2,257) = 35.848, *p* = 0.000, *f*^2^ = 0.279, medium-large effect). Thus, no mediation, but an indirect relationship between additive risk and internalizing symptoms through negative COVID-impact was found. The CRM-analyses, yielded similar results (c’: *R*^2^ = 0.001, adjusted *R*^2^ = − 0.003, *F* (1,258) = 0.222, *p* = 0.638 & b: *R*^2^ = 0.218, adjusted *R*^2^ = 0.212, *F* (2,257) = 35.872, *p* = 0.000, *f*^2^ = 0.279, medium to large effect). Figure 2 shows a graphical representation of these results and Table 3 shows additional statistics.

**Table 3:** Unstandardized (B) and Standardized beta (β) Regression Coefficients, R Squared (R^2^), Significance (p) for all predictors in mediation analysis through separate and hierarchical Regression Models.

Independent variable—dependent variable	B	Beta	*R *^*2*^	*p*	B	Beta	*R *^*2*^	*p*
	ARM				CRM			
A/C Risk^a^—life satisfaction	− 0.103	− 0.187	0.035	0.002**	− 0.012	− 0.167	0.028	0.007**
A/C Risk^a^—COVID-impact	0.458	0.222	0.049	0.000***	0.046	0.169	0.029	0.006**
A/C Risk^b^—life satisfaction	− 0.051	− 0.091	0.223	0.119	− 0.007	− 0.091	0.224	0.104
COVID-impact^b^—life satisfaction	− 0.119	− 0.445	0.223	0.000***	− 0.120	− 0.449	0.224	0.000***
A/C Risk^a^—Internalizing symptoms	0.023	0.056	0.003	0.372	0.002	0.029	0.001	0.638
A/C Risk^b^—internalizing symptoms	− 0.021	− 0.050	0.218	0.376	− 0.003	− 0.051	0.218	0.365
COVID-impact^b^—internalizing symptoms	0.097	0.476	0.218	0.000***	0.097	0.473	0.218	0.000***

Asterisks signify significant effects, * *p* < 0.05, **p < 0.01 & ***p < 0.001.

^a^In a separate regression model.

^b^In a hierarchical regression model, R^2^ of the (combined) hierarchical model.

### Risk clusters in relation to Negative COVID-impact

Hierarchical regression models of risk clusters showed the following: *Individual factors* was a significant cluster, *R*^2^ = 0.125, adjusted *R*^2^ = 0.115, F (3,266) = 12.694, *p* = 0.000. However, within the model, only self-control was a statistically significant predictor. *Parenting* also explained a significant part of the variability (8.4%) in negative COVID-impact, *R*^2^ = 0.084, adjusted *R*^2^ = 0.072, F (5,389) = 7.101, *p* = 0.000. Within parenting factors, negative interaction with mothers and parental responsiveness were significant predictors. *Maternal mental health* was significant as a cluster, *R*^2^ = 0.026, adjusted *R*^2^ = 0.018, F (3,367) = 3.265, *p* = 0.022 (predicting 2.6% variability), with no significant factors within the model. *Paternal mental health* was not significant, *R*^2^ = 015, adjusted *R*^2^ = 0.005, F (3,283) = 1.483, *p* = 0.219, but within the insignificant model, paternal depression was significant. *Family constellation* significantly predicted 2.3% of variability in negative COVID-impact, *R*^2^ = 0.023, adjusted *R*^2^ = 0.015, F (3,385) = 2.979, *p* = 0.031. Within this model the parent–child ratio was the only significant risk factor. Lastly, *Demographic factors* significantly related to negative COVID-impact, *R*^2^ = 0.040, adjusted *R*^2^ = 0.025, F (4,270) = 2.791, *p* = 0.027 (predicting 4% variability). Within the demographic factors, SES and paternal educational level were significant. Table 4 shows additional statistics of abovementioned regression models.

**Table 4:** R^2^, F-change and significance for all hierarchical Regression Models per risk cluster and Unstandardized (B) and Standardized (β) Regression Coefficients, and Significance (p) for all predictors in the models.

Model	Negative COVID-impact		
	B [95% CI]	Beta	p
**Individual factors**			
Educational level	− 0.025 [− 0.325, 0.274]	− 0.010	0.867
Self-control	− 0.275 [− 0.372, − 0.179]	− 0.326	0.000***
Social competence	− 0.104 [− 0.233, 0.024]	− 0.093	0.112
**Parenting factors**			
Negative interaction with P1	0.633 [0.278, 0.988]	0.212	0.001**
Negative interaction with P2	− 0.106 [− 0.394, 0.181]	− 0.044	0.469
Parental responsiveness	− 0.344 [− 0.585, − 0.103]	− 0.142	0.005**
Frequency of joint activity	− 0.015 [− 0.099, 0.069]	− 0.019	0.729
Duration of joint activity	− 0.320 [− 0.648, 0.009]	− 0.103	0.056
**Maternal Mental Health (P1)**			
Stress	0.272 [− 0.069, 0.613]	0.120	0.118
Depression	− 0.260 [− 0.906, 0.385]	− 0.055	0.428
Anxiety	0.318 [− 0.178, 0.813]	0.089	0.209
**Paternal Mental Health (P2)**			
Stress	− 0.219 [− 0.668, 0.230]	− 0.085	0.338
Depression	0.745 [0.007, 1.484]	0.163	0.048*
Anxiety	− 0.024 [− 0.651, 0.604]	− 0.007	0.941
**Family constellation**			
Household size	− 0.710 [− 1.497, 0.078]	− 0.155	0.077
Parent–child ratio	1.763 [0.501, 3.026]	0.247	0.006**
Family composition	− 1.673 [− 4.005, 0.659]	− 0.125	0.159
**Demographic factors**			
SES	− 0.559 [− 0.930, − 0.187]	− 0.190	0.003**
Migration background	− 1.340 [− 7.532, 4.851]	− 0.025	0.670
Educational level P1	− 0.515 [− 1.573, 0.544]	− 0.066	0.339
Educational level P2	0.717 [0.074, 1.359]	0.157	0.029*

Asterisks signify significant effects, *p < 0.05, **p < 0.01 & ***p < 0.001.

The full model of abovementioned significant risk factors significantly explained 22.1% of variability in the negative COVID-impact, *R*^2^ = 0.221, adjusted *R*^2^ = 0.201, F (7, 277) = 11.194, *p* = 0.000. Within the model SES, self-control, negative interaction with mothers and parental responsiveness remained significant (see Table 5).

**Table 5:** Unstandardized (B) and standardized (β) regression coefficients, and significance (p) for all significant predictors in hierarchical regression models.

Variable/riskfactor	B [95% CI]	Beta	p
Parent–child ratio	0.768 [− 0.152, 1.688]	0.089	0.101
SES	− 0.420 [− 0.749, − 0.090]	− 0.146	0.013*
Educational level P2	0.436 [− 0.080, 0.952]	0.096	0.098
Self-control	− 0.310 [− 0.405, − 0.214]	− 0.351	0.000**
Depression P2	0.144 [− 0.352, 0.640]	0.031	0.569
Negative interaction with P1	0.395 [0.040, 0.749]	0.120	0.029*
Parental responsiveness	− 0.281 [− 0.547, − 0.015]	− 0.112	0.039*

Asterisks signify significant effects, *p < 0.05 & **p < 0.01. P1 = mothers, P2 = fathers.

## Discussion

The current study examined whether a stronger negative COVID-impact on youth is related to lower life satisfaction and more internalizing symptoms, and if this relationship is mediated by additive and cumulative risk exposure. Risk exposure was operationalized in four ways; Additive and Cumulative Risk, clusters of risk and the most salient risk factors.

First and foremost, current findings demonstrate that youth who perceive the COVID-impact more negatively report significantly lower life satisfaction and more internalizing symptoms. Although no conclusions can be drawn about causality, the findings are in line with previous studies showing negative associations between COVID and emotional/psychological wellbeing^2,3^. Mental health issues like internalizing symptoms in childhood and adolescence can be related to negative (mental health) outcomes in (young) adulthood, such as a higher likelihood to suffer from mental health problems/meet criteria for *DSM-(I)V* diagnoses and more^42^. Possibly suggesting that early intervention for youth suffering from the COVID-19 restrictions is also important in view of potential long-term consequences. Additionally, it was found that the negative COVID-impact was larger for girls than for boys and worsened with age. This could be because older youth have more to lose compared to younger youth because peers become more important^5^, while strictness of COVID restrictions increase with age^1^.

Given the significant relationship between COVID-impact and youth well-being it is imperative to better understand how risk exposure accounts for the negative effects on youth’s lives. It was hypothesized that the relationship between both the sum (ARM) and accumulation (CRM) of risk factors and lower psychological well-being was reinforced by negative COVID-impact. During the intelligent lockdown, both ARM and CRM risk exposure were directly related to lower life satisfaction. The relationship between ARM and CRM with life satisfaction was indeed mediated through negative COVID-impact on youth. Secondly, ARM and CRM also affect internalizing symptoms among youth indirectly through negative COVID-impact, but there was no mediated relationship between the risk models and internalizing symptoms through negative COVID-impact. Nor did ARM and CRM directly influence internalizing symptoms in youth. This finding is unexpected, but may be explained by a delayed effect of cumulative risk on youth. Possibly, there are no (measurable) immediate and concurrent adverse effects of (family) stressors on youth psychosocial development, but there are longitudinal effects that only emerge over time^43^. Lastly, the ARM and CRM models seem to be equally useful for mapping the negative COVID-impact’s effect on youth wellbeing, albeit indirectly. Perhaps examination of clusters and individual factors first, and then including only significant risk factors that emerged in the ARM/CRM analyses would yield different results.

Clusters of risk and individual risk factors were also studied. This showed that ‘Individual factors’, ‘Parenting’, ‘Maternal mental health’ and ‘Demographic factors’, play an important role in the COVID-impact as a cluster and ‘Paternal mental health’ and ‘Family constellation’ did not. Within youth individual factors, self-control was a particularly salient factor. This is likely an important factor, since self-control is not only about regulating behavior and controlling impulses, but entails regulation of emotions and thoughts as well (i.e. how the pandemic is dealt with emotionally^14^). Among the parenting factors, youth-reported negative interaction with mothers and low parental responsiveness were particularly important factors in the negative COVID-impact for youth, corresponding with findings that positive interaction/relationships with parents and parental responsiveness are important to youth wellbeing and outcomes^17,18^. Although ‘Maternal mental health’ was an important cluster, the separate maternal risks (depression, anxiety and stress) had no particularly important part in this, suggesting that the full picture of maternal mental health is important. From the paternal mental health cluster, depression was an important factor. Paternal depression was previously directly related to internalizing behaviors among youth, whereas maternal depression did not^44^. Among the family constellation factors, an unfavorable parent–child ratio remained as only relevant factor, possibly indicating that the more children outnumber parents in a given family, the more difficult the pandemic becomes to cope with. For the demographic factors, a lower SES and a higher level of paternal education was related to a stronger negative COVID-impact. As previously demonstrated, it seems that lower SES families are more likely to experience negative consequences of the lockdown^45^, which is in line with findings supporting the poverty related-stress model^46^. Opposite this, a higher paternal educational level seems related to a stronger negative COVID-impact among youth. We can only contemplate about the interpretation of this finding. Perhaps in families with fathers with a higher educational level the typical home situation changes more compared to other families. They are more likely to start working from home, because their jobs allow them to do so more often, compared to lower educated fathers in more practical work fields who may continue as usual.

Lastly, when the relevant separate risk factors from the risk clusters were studied in relation to the negative COVID-impact, low SES, low self-control, negative interaction with mothers and parental responsiveness remained as most salient risk factors.

### Limitations, strengths and future directions

The current study has a cross-sectional design, meaning no conclusions can be drawn regarding directionality of effects. Secondly, because existing data was utilized from a study of which the data collection coincided with the lockdown—an unexpected situation—extensive examination of relevant risk factors could not be conducted prior to starting data-collection. Third, the data exists in large part of sibling data, which is uncontrolled for in the analyses. Lastly, the research sample lacks diversity in terms of ethnicity, SES and educational level, reducing the external validity and resulting in lower variance reducing statistical power.

Nonetheless, the current study makes use of participants’ natural environment, which in turn increases the importance of the findings. Another strength is the use of standardized, well-researched instruments. Lastly, the study has an innovative character as it differentiates between Additive and Cumulative Risk.

For follow-up examination of risk exposure in the context of COVID-19 overtime, a longitudinal design would offer the potential to examine directionality of effects. Prior research showed that already vulnerable families (due to risk accumulation) are more likely to be less resilient^11^. The approach of the current study to risk exposure also holds value for studying vulnerable, and clinical populations.

## Conclusion

In sum, a more negative experience of the COVID-19 pandemic and restrictions is clearly related to decreased life satisfaction and increased internalizing symptoms among youth (9–18 years). The negative impact is larger for girls and increases with age. Negative COVID-impact is related to additive, as well as cumulative risk exposure. There is an indirect relationship between ARM and CRM and internalizing symptoms, but no direct relationship. The relationship between ARM and CRM and life satisfaction is mediated through the negative COVID-impact. The Additive and Cumulative Risk Models prove equally valid for predicting variability in the negative COVID-impact and in life satisfaction. Significant clusters of risk factors (and significant risk factors within these clusters) that related to a stronger negative COVID-impact are ‘Demographic factors’ (low SES and high paternal educational level), ‘Individual factors’ (low self-control), ‘Maternal mental health’ (as a cluster) and ‘Parenting’ (negative mother–child interaction and low parental responsiveness). Furthermore, unfavorable parent–child ratio and paternal depression are significant separate risk factors. The most salient individual risk factors remaining from abovementioned clusters and factors are low SES, low self-control, negative interaction with mothers and low parental responsiveness.

Abovementioned factors may yield relevance for timely identification of at-risk youth, including assessments of risk not only pertaining to youth but also to their environment; the family. Longitudinal research is essential to explore the long-term effects of COVID-19 restrictions on youth development and its effects on vulnerable populations.

## Supplementary Information

Supplementary Information.

## Data Availability

The dataset analyzed during the current study are not publicly available at this point because it is part of a longitudinal study of a PhD-student. Data are available from the corresponding author on reasonable request.
