# Supplementary material for: Examination of risk exposure models during COVID-19 in relation to youth life satisfaction and internalizing symptoms
Source: Sci Rep. 2022 Sep 28;12:16252. doi: 10.1038/s41598-022-20661-2 (PMC9518947; doi:10.1038/s41598-022-20661-2)
Supplement: Supplementary file 1 — Supplementary Information. [file 41598_2022_20661_MOESM1_ESM.docx]

**Appendix A**

**Negative COVID-impact original Questionnaire with translation**

**CORONAVIRUS**

De volgende vragen gaan specifiek over de situatie rondom het Coronavirus (COVID-19). Het coronavirus zorgt bij veel mensen voor onzekerheid. Er zijn grote veranderingen, bijvoorbeeld omdat de scholen dicht zijn en je niet meer veel vrienden tegelijkertijd mag afspreken. De volgende vragen gaan over hoe deze situatie voor jou is.

[The following questions specifically address the Coronavirus (COVID-19) situation. The Coronavirus brings insecurities for many people. There are substantial changes, for example the closures of schools or not being able to meet up with multiple friends at once. The following questions pertain to your personal experience of this situation. ]

**V31 Corona-items**

Geef aan in hoeverre onderstaande uitspraken in DE AFGELOPEN TWEE WEKEN. voor jou wel of niet kloppen

[ Indicate to what extent the statements below did or did not apply to you for THE PAST TWO WEEKS. ]

1. Ik ben bang dat ik zelf of mensen om mij heen met het Coronavirus besmet wordt/worden.

[ I’m afraid that I or the people around me will get infected with the Coronavirus. ]

1. Door de Corona crisis is er meer ruzie binnen ons gezin.

[ Because of the COVID-crisis there are more conflicts within the family ]

1. Door de Corona crisis doe ik meer activiteiten samen met mijn ouder(s).

[ Because of the COVID-crisis, I do more activities with my parent(s). ]

1. Door de Corona crisis maak ik me zorgen over mijn schoolwerk.

[ Due to the COVID-crisis, I worry about my schoolwork. ]

1. Ik vermaak me prima nu ik door de Corona crisis meer thuis ben.

[ I’m enjoying myself now that I’m more often at home due to the COVID-crisis. ]

1. Ondanks de Corona crisis is de sfeer bij ons thuis goed.

[Despite the COVID-crisis, the atmosphere with us at home is good. ]

1. Vanwege de Corona crisis slaap ik slecht.

[ Because of the COVID-crisis, I’m sleeping badly. ]

1. Ondanks de Corona crisis blijf ik actief (o.a. huiswerk maken, wandelen, sporten).

[ Despite the COVID-crisis, I remain active (e.g., doing homework, going for walks, exercising). ]

1. Ondanks de Corona crisis houd ik actief contact met mijn vrienden.

[ Despite the COVID-crisis, I actively keep in touch with my friends. ]

Antwoordcategorieën: [Answer categories:]

1 Helemaal oneens [Completely disagree]

2 Oneens [Disagree]

3 Neutraal  [Neutral]

4 Eens [Agree]

5 Helemaal eens [Completely agree]
